# Association between periodontal and geriatric health: a cross-sectional study

**DOI:** 10.1007/s00784-026-06938-0

**Published:** 2026-05-30

**Authors:** Madeleine Larissa Müller, James Deschner, Michael Mohr, Philipp C. Mildenberger, Roland Hardt, David Kiramira

**Affiliations:** 1 Department of Periodontology and Operative Dentistry, University Medical Centre of the Johannes Gutenberg University, Mainz, Germany; 2 Department of Prosthodontics and Dental Materials Science, University Medical Centre of the Johannes Gutenberg University, Mainz, Germany; 3 Geriatric Department, University Medical Centre of the Johannes Gutenberg University, Mainz, Germany; 4 Institute of Medical Biostatistics, Informatics and Epidemiology, University Medical Centre of the Johannes Gutenberg University, Mainz, Germany; 5 Research Center for Immunotherapy (FZI), University Medical Center of the Johannes Gutenberg University, Mainz, Germany

**Keywords:** Geriatric assessment, Periodontal diseases, Tinetti test, POMA, Performance-oriented mobility assessment, Mini mental state examination

## Abstract

**Objectives:**

This cross-sectional study investigated the association between periodontal status and geriatric health parameters, a relationship that has received little attention in research to date.

**Materials and methods:**

The periodontal health of 51 geriatric patients was assessed based on probing pocket depth (PPD), clinical attachment level (CAL), bleeding on probing (BOP) and approximal plaque index (API). General health was evaluated using the Tinetti Test, Mini-Mental State Examination (MMSE), handgrip strength test and the Barthel Index. Oral hygiene habits were evaluated using a structured questionnaire.

**Results:**

CAL was significantly associated with patients fall risk determined by Tinetti Test. An increase in CAL corresponded with a decline in balance and gait (*p* < 0.05). BOP and API were also significantly associated with Tinetti Test (*p* < 0.05). Moreover, a decrease in CAL was significantly associated with an increased handgrip strength (*p* < 0.05). A significant inverse correlation was also found between API and MMSE (*p* < 0.05). The present study revealed no significant association between Barthel Index and periodontal parameters.

**Conclusions:**

The study suggests that a decline in periodontal health is associated with a deterioration in general health in older age, with the Tinetti Test potentially serving as a valuable geriatric parameter.

**Clinical relevance:**

These findings may promote the development of targeted preventive strategies focused on the periodontal health requirements of this hospitalised patient group.

**Supplementary Information:**

The online version contains supplementary material available at 10.1007/s00784-026-06938-0.

## Introduction

 The global geriatric population, defined as individuals aged 65 years and older, is projected to comprise approximately 16% of the global population by 2025 according to United Nations estimates [1]. With increasing age, the progression of physical frailty and the accumulation of multiple comorbidities can substantially compromise quality of life. In Germany, an estimated 13% of older adults are classified as frail, a condition associated with elevated healthcare costs, a higher likelihood of institutionalisation, and increased mortality rates [2]. Consequently, the challenges associated with prolonged longevity have become an increasingly critical concern for public health systems.

Elderly frailty can also affect an individual’s ability to maintain adequate oral hygiene. As age advances, patients often become less aware of food debris or plaque accumulation and may encounter difficulties performing proper oral hygiene routines due to age-related motor or cognitive decline [3]. Accordingly, the incidence of bacterial plaque-associated diseases, such as periodontitis and caries increase with age [4], often accompanied by tooth loss, reduced masticatory function, and a decline in oral health-related quality of life [5]. Furthermore, the prevalence and progression of age-associated systemic conditions, such as diabetes, Alzheimer’s disease, and coronary heart diseases, are strongly linked to the presence and severity of periodontitis [6]. The assessment of functional abilities, cognition, mental health, motor skills and nutrition, among other factors, is essential for accurate diagnosis, prognosis and management of oral diseases. For instance, the evaluation of handgrip strength may indicate the need for assistance with oral hygiene procedures or the use of adapted brushing instruments and techniques. Similarly, evidence of malnutrition may reveal underlying dysphagia or masticatory difficulties, that are not immediately apparent. In addition, medications commonly prescribed to elderly patients can contribute to oral complications, for example, bisphosphonates and other antiresorptive agents may induce mucosal ulcerations and osteonecrosis of the jaw [7]., while immunosuppressive drugs have been associated with gingival enlargement and altered periodontal inflammation [8, 9]. The study aimed to investigate the bidirectional relationship between periodontal health and geriatric assessments.

## Materials and methods

### Study design

The present cross-sectional study received approval from the Rhineland-Palatinate State Medical Association Ethics Committee before its execution (No. 2022–16574). The implementation, evaluation and documentation of this study followed the requirements of Good Clinical Practice (GCP) and Good Epidemiological Practice (GEP), as well as the ethical principles stated by the Declaration of Helsinki as revised in 2024. It was conducted between January 2022 and September 2023 at the Geriatric Clinic of the Mainz University Medical Centre.

### Patients’ inclusion

During the study period, a total of 386 patients were treated in the geriatric department. Of these, 51 provided informed consent to participate and could be included in the study. Due to the nature of the assessment, only patients who agreed to undergo the oral examination, and for whom it could be sufficiently performed at the bedside, were included. Regarding the clinical context, participants were predominantly hospitalised due to acute medical conditions. These included admissions via the emergency department and post-operative care (e.g., following fractures), as well as cases of acute deterioration in general health.

### Oral assessments

Patients were examined in the geriatric ward after providing informed consent and receiving a detailed explanation of the examination procedures. All oral examinations were performed at the patient’s hospital bed and lasted usually 30 min, with a maximum duration of 60 min. To ensure consistency, all clinical examinations were conducted by a single examiner. Initially, a general and specific anamnesis was collected. This was followed by a comprehensive dental examination, during which the number of teeth, the presence of dental and mucosal lesions, the type and condition of dentures, the presence and number of implants, and the number of occluding teeth were recorded. The patients’ periodontal status was assessed by the following parameters using a periodontal probe (UNC-15, Hu- Friedy, Chicago, IL, USA) and furcation probe (Nabers #2 N, Hu-Friedy, Chicago, IL, USA): clinical attachment level (CAL), probing pocket depth (PPD), bleeding on probing (BOP), approximal plaque index (API), and gingival recession (GR), measured at six sites per tooth. In addition, the tooth mobility and furcation involvement were evaluated. Due to the absence of periapical radiographies and x-ray equipment at the geriatric unit, the clinical diagnosis of periodontitis was based on the presence of two or more interproximal sites with BOP, PPD ≥ 4 mm and CAL ≥ 5 mm on different teeth [10]. 

Supplementary to the clinical examination, a questionnaire evaluating patients’ general oral hygiene conditions was applied. The questionnaire contained questions regarding the use of dentures, the performance of self-oral hygiene, the frequency of oral hygiene practices, instruments used for oral hygiene, symptoms of xerostomia, chewing capacity and pain.

### Geriatric assessments

Data from the comprehensive geriatric assessment (CGA), including the Tinetti Test, which is also known as Performance-Oriented Mobility Assessment (POMA), the Mini-Mental State Examination (MMSE), the Barthel Index and handgrip strength measurements, were retrieved from the geriatric medical report. Additionally, care level and diet were documented.

The Tinetti Test is a reliable and valid clinical tool for assessing balance and gait in elderly individuals. The assessment encompasses a range of balance measures, including static, dynamic, reactive, and anticipatory balance, as well as ambulation and transfer ability. The test can be administered within a time frame of approximately five minutes, and it is frequently incorporated into CGA [11]. The MMSE is a widely used screening tool for assessing the cognitive abilities of older adults. Owing to its clearly structured design and broad applicability, it enables an objective evaluation of mental performance on a 30-point scale [12]. Independence in activities of daily living is assessed using the Barthel Index. It comprises ten basic activities, such as eating, bathing, dressing, maintaining personal hygiene, using the toilet, controlling the bladder and bowels, transferring (e.g. from bed to chair), mobility, and stair climbing. Each activity can be awarded up to ten points, resulting in a maximum total score of 100 points. Higher scores indicate greater functional independence [13]. Handgrip strength is measured to assess general physical performance. The maximum force of the dominant hand is recorded using a dynamometer. Handgrip strength correlates positively with general muscle strength and negatively with the risk of falls, bone fractures and overall mortality [14]. The care level refers to the classification of individuals requiring care due to health impairments or disabilities into different categories based on the extent of their needs. In the German care system, five care levels (1 to 5) are defined, with care level 1 representing the lowest and care level 5 representing the highest level of support required. A higher level of care is indicative of a more comprehensive range of care services [15].

### Statistical analysis

All statistical analysis were performed using the ‘SPSS’ software (Version 29.0.2.0., Nordhastedt, Germany). Descriptive and regressive data were expressed as means and standard errors. Linear regression analysis with robust variance estimation was used to determine the associations between continuous and categorical influencing variables and the periodontal parameters. The statistical level of significance was set at p < 0.05 and the regression coefficient was shown to indicate the strength and direction of the relationship between the independent and dependent variable.

## Results

### Geriatric patients

A total of 51 patients admitted to the geriatric department were included in the study. Patients were hospitalised in the geriatric unit between July 2022 to September 2023, with an approximate stay of two weeks. As part of this study, key demographic and care-related characteristics of the study population were systematically recorded and analyzed.

At the time of the examination, the mean age of the patients was 83.75 ± 6.13 years, ranging from 69 to 99 years. Table 1 shows the general demographic characteristics of the included patients. Regarding gender distribution, there was a predominance of female patients with 32 (62.7%) of female respondents, while 19 (37.3%) were male. Assessment of patients’ care level revealed that 27 (52.9%), 5 (9.8%), 10 (19.6%), 8 (15.7%) and 1 (2.0%) patients had no impairment of independence (care level 0), minor impairment (care level 1), significant impairment of independence (care level 2), severe impairment of independence (care level 3), and severe impairment of independence with special requirements for nursing care (care level ≥ 4), respectively. Only 3 patients (5.9%) suffered from swallowing difficulties, whereas 48 (94.1%) had no such difficulties. According to the WHO classification, the BMI was collected and included 3 individuals (5.9%) with BMI < 18.5, who were classified as underweight. Nineteen patients (37.3%) had a BMI within the normal range (18.5 ≤ BMI ≤ 25). Furthermore, 23 (45.1%) with a BMI between 25 and under 30 were categorized as overweight while 6 patients (11.8%) had a BMI of ≥ 30 and were therefore categorized as obese (Table 1). Additional outcomes of the present section are presented in Table 1.

**Table 1:** Characteristics of the study population, sociodemographic

Variables		*n* (%)	Mean ± SD
Sex	Female	32 (62.7)	-
	Male	19 (37.3)	
Age	All study patients	51 (100)	83.75 ± 6.13
	Female	32 (62.7)	84.37 ± 5.99
	Male	19 (37.3)	82.68 ± 6.39
Marital status	Single	8 (15.7)	-
	Married	18 (35.3)	
	Widowed	25 (49.0)	
Living environment	Alone	31 (60.8)	-
	Alone with help	20 (39.2)	
Care level	0 (No impairment of independence)	27 (52.9)	-
	1 (Minor impairment of independence)	5 (9.8)	
	2 (Significant impairment of independence)	10 (19.6)	
	3 (Severe impairment of independence)	8 (15.7)	
	≥ 4 (Severe impairment of independence with special requirements for nursing care)	1 (2.0)	
Body Mass Index (BMI)	Underweight (BMI < 18.5)	3 (5.9)	25.60 ± 5.28
	Normal Weight (18.5 ≤ BMI < 25)	19 (37.3)	
	Overweight (25 ≤ BMI < 30)	23 (45.1)	
	Obesity (BMI ≥ 30)	6 (11.8)	
Dysphagia	Yes	3 (5.9)	-
	No	48 (94.1)	
Diet	Pureed diet	1 (2.0)	-
	Soft diet	6 (11.8)	
	Wholefood diet	44 (86.3)	

### Oral and periodontal parameters

The average number of teeth per patient was 14.9 (Table 2). Of these, 22 patients still had their own teeth, 25 patients had a combination of own teeth and dentures, and only 4 patients were fitted entirely with dentures. Chewing ability, xerostomia and general oral pain was surveyed, showing that 44 out of 51 patients were able to chew physiologically, 36 patients suffered from xerostomia, and only 1 patient reported oral pain (Table S1).

In total, 456 of 762 teeth were affected by periodontal disease in general, while 306 teeth were unaffected, representing 59.84% of the study population. Patients were classified into stages I-IV to categorise the severity and extent of periodontal disease, with no patients classified as stage I. Stage II periodontitis was diagnosed in 8 patients (15.7%), stage III affected 16 patients (31.4%), and 27 subjects were classified as stage IV. Furthermore, the mean CAL was 3.76 mm, mean BOP was 77.0% (± 26.38), mean PPD was 2.58 mm (± 0.54), and the mean API was 81.92% (± 27.48). Tooth mobility was not found in 9 patients (17.6%), while 15 individuals (29.4%) had mobility degree I, 19 (37.3%) mobility degree II and 8 patients mobility degree III. The mean gingival recession was 1.18 mm (± 0.92) (Table 2). In Table 2 more detailed findings of the present section can be found.

**Table 2:** Characteristics of the study population, periodontal aspects

Variables		*n* (%)	Mean ± SD
Number of teeth	-	762 (100)	14.94 ± 8.2
Number of healthy teeth	-	306 (40.16)	6 ± 6.36
Number of periodontally affected teeth	-	456 (59.84)	8.94 ± 6.11
Staging	I	0 (0)	3.37 ± 0.75
	II	8 (15.7)	
	III	16 (31.4)	
	IV	27(52.9)	
Mean Clinical Attachment Level (CAL)	-	-	3.76 ± 1.45
Bleeding on Probing (BOP)	-	-	77.0 ± 26.38
Mean Probing Depth (PPD)	-	-	2.58 ± 0.54
Mean Approximal Plaque Index (API)	-	-	81.92 ± 27.48
Tooth mobility	Mobility degree 0	9 (17.6)	1.51 ± 0.967
	Mobility degree I	15 (29.4)	
	Mobility degree II	19 (37.3)	
	Mobility degree III	8 (15.7)	
Mean Recession	-	-	1.18 ± 0.92
Max furcation level	-	-	0.63 ± 0.93
Localised Periodontitis	-	9 (17.6)	-
Generalized Periodontitis	-	42 (82.4)	-

### Geriatric parameters

The mean MMSE score, as a measure of cognitive function, was 25.43 (± 3.96), including 27 patients (52.9%) with no cognitive impairment, 20 patients exhibiting mild impairment, 3 individuals (5.9%) presenting moderate impairment, and only one patient (2.0%) showing severe cognitive impairment. The Barthel Index was used to assess self-help capability and independence, with 3 patients (5.9%), 20 patients (39.2%), 23 patients (45.1%), and 2 patients (3.9%) demonstrating mild or no, moderate, severe, and total dependency, respectively. Handgrip strength was assessed as a measure of muscle performance. Sixteen patients (31.4%) had a grip power of ≥ 25 kg, whereas 35 patients (68.6%) demonstrated a weak grip strength. The Tinetti Test revealed that 6 (11.8%), 5 (9.8%), and 40 (78.4%) patients had a low, moderate, and high risk of falling, respectively (Table 3).

**Table 3:** Characteristics of the study population, geriatric assessments

Variables		*n* (%)	Mean ± SD
Mini-Mental State Examination (MMSE)	No impairment (30 − 27 points)	27 (52.9)	25.43 ± 3.96
	Mild cognitive impairment (26 − 21 points)	20 (39.1)	
	Moderate impairment (20 − 10 points)	3 (5.9)	
	Severe impairment (< 10 points)	1 (2.0)	
Barthel Index	Independent (100 − 90 points)	3 (5.9)	50.49 ± 19.42
	Mild dependency (89 − 75 points)	3 (5.9)	
	Moderate dependency (74 − 50 points)	20 (39.2)	
	Severe dependency (49 − 25 points)	23 (45.1)	
	Total dependency (< 25 points)	2 (3.9)	
Handgrip strength	Normal strength (≥ 25 kg)	16 (31.4)	16.07 ± (8.09)
	Weak grip strength, risk of sarcopenia (< 25 kg)	35 (68.6)	
Tinetti Test	Low fall risk (≥ 24 points)	6 (11.8)	12.82 ± (7.26)
	Moderate fall risk (19–23 points)	5 (9.8)	
	High fall risk (≤ 18 points)	40 (78.4)	

### Correlation of CAL with geriatric parameters, oral hygiene and demographic characteristics

Regarding CAL, a highly significant association was observed with fall risk, as described by the Tinetti Test. According to the regression coefficient, an increase in the Tinetti index was significantly associated with a decrease in CAL. (ß = -0.07, *p* < 0.05). Significant correlations were also found between the handgrip strength and CAL (ß = -0.04, *p* < 0.05), indicating that deterioration in periodontal condition was associated with reduced muscular performance. No significant correlation was observed between CAL and either Barthel Index or MMSE (Table 4). Furthermore, care level ≥ 4 showed a significant correlation with periodontal condition as well (ß = -0.65, *p* < 0.001) (Table S2.1). Concerning oral hygiene, CAL was found to be significantly correlated with the brushing frequency, with higher frequency linked to lower CAL values (1/d ß = -4.15, 2/d ß = -4.35, 3/d ß = -4.15, *p* < 0.001). In terms of staging, stages III and IV were significantly associated with increased CAL relative to stage II (Stage III: ß = 0.28 *p* < 0.001, Stage IV: ß = 0.32, *p* < 0.001), respectively (Table S2.2).

**Table 4:** Regression results analyzing the association of CAL with geriatric indices

Correlated variable	Regression coefficient	Std-Error	*p*-value
Tinetti Test	-0.072	0.029	0.015*
Handgrip strength	-0.041	0.207	0.046*
Barthel Index	-0.010	0.011	0.345
MMSE	0.023	0.276	0.4

### Correlation of PPD with geriatric parameters, oral hygiene and demographic characteristics

PPD was not found to be significantly linked to any of the investigated geriatric assessments, while the correlation of Tinetti Test only marginally exceeded the predefined significance level (Table 5). Care level ≥ 4 (ß = -0.59, *p* < 0.05), diet (Mixed food: ß = 1.3, *p* < 0.05, Wholefood: ß = 0.35, *p* < 0.001), and most of the oral hygiene and oral health parameters, excluding the dysphagia index and xerostomia, were significantly correlated with PPD (Table S3.1 and S3.2). A strong inverse association was observed between brushing frequency and PPD, indicating reduced probing depths with increasing brushing frequency (1/d ß = -2.59, 2/d ß = -2.84, 3/d ß = -2.59, *p* < 0.001). The absence of oral pain (ß = 1.01, *p* < 0.001) and an increment of PPD across Stage II to IV periodontitis (Stage III: ß = 0.98 *p* < 0.001, Stage IV: ß = 1.4, *p* < 0.001) also displayed statistical significance (Table S3.2).

**Table 5:** Regression results analyzing the association of PPD with geriatric indices

Correlated variable	Regression coefficient	Std-Error	*p*-value
Tinetti Test	-0.039	0.021	0.064
Handgrip strength	-0.015	0.017	0.37
Barthel Index	-0.005	0.007	0.475
MMSE	-0.007	0.021	0.73

### Correlation of BOP with geriatric parameters, oral hygiene and demographic characteristics

BOP showed a significant association with the Tinetti score, with decreasing BOP corresponding to an improvement in balance and gait performance as described by the fall risk (ß = -1.08, *p* < 0.05). BOP could not be significantly associated with further of the geriatric assessments (Table 6). Significant relations can be described regarding brushing frequency (2/d ß = -25.63, 3/d ß = -24.93, *p* < 0.001), as well as xerostomia (ß = 16.34, p = < 0.05) and the chewing capacity (ß = *p* < 0.001). Oral hygiene dependency was positively correlated with BOP (ß = 16.65, *p* < 0.05). Additionally, advanced periodontal stages were significantly associated with BOP (Stage III: ß = 42.63, *p* < 0.001, Stage IV: ß = 48.17, *p* < 0.001) (Table S4.2). Table S4.1 presents additional correlations between BOP and sociodemographic variables.

**Table 6:** Regression results analyzing the association of BOP with geriatric indices

Correlated variable	Regression coefficient	Std-Error	*p*-value
Tinetti Test	-1.075	0.509	0.034*
Handgrip strength	-0.213	0.521	0.689
Barthel Index	-0.209	0.179	0.245
MMSE	-0.734	0.644	0.254

### Correlation of API with geriatric parameters, oral hygiene and demographic characteristics

API demonstrated a significant inverse correlation with the Tinetti Test. Accordingly, higher Tinetti scores were significantly associated with lower API values (ß = -0.875, *p* < 0.05). Decreasing API values were significantly associated with higher MMSE scores (ß = -1.540, *p* < 0.05), suggesting a link between oral hygiene status and cognitive function (Table 7). API was also shown to be significantly linked to two demographic variables addressing care level ≥ 4 and diet, respectively (care level ≥ 4: ß = 19.41, Mixed food: ß = -22.5, *p* < 0.05, Wholefood: ß = -17.89, *p* < 0.001) (Table S5.1). In consideration of the oral hygiene, the use of an electric toothbrush in comparison to a manual device leads to a statistically relative decrease in the API (ß = -16.66, *p* < 0.05) (Table S5.2). Except for dysphagia index and xerostomia most of the oral hygiene and oral health parameters provided a significant prediction (Table S5.1 and S5.2).

**Table 7:** Regression results analyzing the association of API with geriatric indices

Correlated variable	Regression coefficient	Std-Error	*p*-value
Tinetti Test	-0.875	0.436	0.045*
Handgrip strength	0.093	0.471	0.843
Barthel Index	0.029	0.157	0.855
MMSE	-1.540	0.668	0.021*

## Discussion

The present study investigated the association between periodontal parameters and geriatric conditions such as motor skills, cognitive function and self-help capability. The results showed that periodontal parameters were correlated with functional limitations and cognitive impairment such as reduced physical strength, balance deficits, and increased fall risk. Our findings suggest that a decline in periodontal health is associated with a deterioration in general health in older age, and that the Tinetti Test may serve as a valuable geriatric assessment tool.

The Tinetti Test is a performance-based assessment commonly used in elderly populations to evaluate mobility problems [11]. In the present study, a significant decrease in CAL was observed in patients with higher Tinetti scores. This suggests that limited mobility may have an indirect negative impact on periodontal health, possibly due to a reduced ability to perform adequate oral hygiene or an increased reliance on accessing treatment facilities. Previous studies that focused primarily on Tinetti Assessment Battery for Gait and Balance (TAB) have shown a correlation between tooth loss and a decline in motor function [16]. However, to date, we are aware of no studies that have examined the correlation between Tinetti Test and periodontal parameters, which makes this study unique. Furthermore, a significant decline in BOP and API scores was observed alongside an improvement in Tinetti scores, likewise indicating a correlation between deteriorating gingival condition, as impaired coordination is potentially leading to poor oral-hygiene ability. Although PPD was marginally below the significance threshold, a comparable hypothesis can be drawn for this parameter. This discrepancy may be attributed to the relatively small sample size (*n* = 51), the vulnerability of the patient group or potential measurement variability. Further studies involving larger cohorts and longitudinal designs are warranted in order to clarify the potential influence of the fall risk and PPD.

The Handgrip strength is defined as a measure of the maximal force produced by both the intrinsic and extrinsic hand muscles during flexion of the hand [17]. Aligning with prior research, study outcomes revealed a significant correlation between a decrease in measured handgrip strength of the patients examined and CAL [18, 19].

MMSE is a widely used screening tool for assessing cognitive function in older adults. Its standardised format and international applicability make it a reliable 30-point scale for evaluating mental performance [12]. In the present study, significant associations were observed between cognitive impairment and API. These findings are consistent with previous reports and a recent systematic review demonstrating a comparable relationship between periodontal deterioration and cognitive impairment [20].

No significant associations were observed between periodontal parameters and the Barthel Index in the present study. Although initial trends could be discerned within the data, the magnitude and direction of these correlations could not be determined with statistical significance. Future research should further explore the potential influence of functional independence on periodontal health outcomes.

Higher levels of care were significantly associated with greater CAL, as well as increased PPD and API values. The majority of patients in this study were classified as care level 0 or 2, indicating a generally low need for assistance within the study population.

A lower frequency of toothbrushing was correlated with periodontal deterioration, reflected by higher CAL, PPD, BOP and API values. Conversely, geriatric patients who brushed their teeth several times per day exhibited significantly better periodontal parameters across all indices. These findings are consistent with previous research demonstrating a strong relationship between declining oral hygiene practices and deteriorating periodontal health [21]. The questionnaire additionally assessed patient’s oral hygiene practices, indicating that the use of electric toothbrushes was associated significantly lower PPD and API values. This corroborates prior evidence of improved periodontal health in elderly individuals using electric devices [22].

Within the present study population, a potential correlation between the deterioration of the periodontal condition and patients requiring care was observed, as reflected by increased values of PPD, BOP and API and underlined by a high level of statistical significance. In the geriatric unit, dietary management typically involves adapting nutritional intake to meet elevated protein and micronutrient requirements, while also accommodating chewing and swallowing difficulties by adjusting food consistency. Patients consuming mixed or solid diets exhibited lower indicators of periodontal disease, including reduced BOP, PPD and API values. Findings from previous studies in non-geriatric populations suggest that dietary patterns and nutritional intake may reflect general health status and are associated with more favorable oral condition [23].

Reduced chewing capacity is frequently associated with compromised dental health, as age-related muscle atrophy and diminished motor skills may impair effective oral hygiene. This can promote the accumulation of bacterial plaque and heightened risk of periodontal diseases. In line with earlier research [24], our findings indicated a relationship between reduced chewing capacity and increased periodontal parameters. As the data were obtained through self-reported questionnaires, the predictive strength of these results may be limited. Therefore, the present study provides a specific perspective on the oral health of hospitalised geriatric patients, suggesting that advancing age and frailty are associated with worsening periodontal conditions. A positive correlation between increasing frailty and periodontal deterioration has been demonstrated in previous studies using the frailty index alongside periodontal parameters [16].

Impaired mobility in geriatric patients reflects a complex interplay between age-related physiological changes and multiple chronic diseases. On one hand, ageing is accompanied by a decline of muscle mass and strength, frequently culminating in sarcopenia, which directly impairs mobility and balance [25]. On the other hand, prevalent chronic conditions in the elderly, such as osteoarthritis and obesity, contribute to joint pain and stiffness, further restricting mobility [26]. Neurodegenerative disorders with high prevalence in the elderly, such as Parkinson’s disease, are known to cause motor dysfunction, adversely affecting both balance and coordination [27].

Chronic inflammation associated with periodontal disease may accelerate senescence and contribute to the development of other age-related morbidities [28]. Periodontitis is linked to the overexpression of biomarkers indicative of altered physiological aging, as well as declines in physical function and increased mortality among older people [29]. The severity of periodontitis, compounded by comorbid conditions such as overweight and cardiometabolic disorders, further promotes its association with premature ageing [30]. This bidirectional relationship between aging, its related comorbidities, and periodontitis mutually contributes to the progressive deterioration of both oral and overall physical health in elderly patients [31, 32].

Elderly frailty and progressive functional impairment are not only linked to age-related pathologies but also to poor oral health [33]. The gradual age-related decline in oral function, due to factors such as tooth loss, insufficient oral hygiene, ill-fitting dental prostheses, and impaired chewing ability, among other factors that may compromise oral functions, have been conceptualized as ‘oral frailty’ [34]. Moreover, oral frailty interacts with physical frailty, systemic inflammation, and psychosocial factors affecting self-esteem related to smiling, chewing, and speech. Functional disability and compromised neuromotor functions have been associated with a decrease in number of teeth, reduced masticatory capacity and poorer oral health-related quality of life [35]. Management of oral frailty through professional oral health care monitoring and interventions such as mastication exercises, including mouth-opening, tongue pressure and prosodic training, has been shown to significantly enhance oral hygiene and reduce the severity of oral frailty [36].

These findings emphasize the need for surveillance and prevention strategies targeting oral health needs in the elderly population. A nursing home-based intervention focused on geriatric oral health showed that providing monthly professional oral care alongside the training and motivation of caregiving staff led to significant improvements in residents’ oral hygiene and caries status. Furthermore, the program enhanced caregivers’ awareness regarding the importance of maintaining oral hygiene in institutionalized older adults [15]. Therefore, both oral health education alongside practical oral hygiene training for both the elderly and their caregivers may therefore be considered as effective strategies to prevent further deterioration of oral health [15].

Despite its valuable findings, the present study has several limitations. The hospital-based cohort may restrict the generalisability to community-dwelling elderly populations. Additional constraints include the study design, small sample size, cross-sectional study approach, and an unbalanced gender distribution. Radiographic assessments were not performed, longitudinal data were unavailable, and periodontal grading could not be conducted. Furthermore, this patient population presents unique challenges that warrant further investigation in future studies. Nevertheless, the integration of geriatric and periodontal parameters within an interdisciplinary study design has rarely been implemented to this extent, highlighting the unique approach of the present study. These findings further substantiate the expanding body of evidence linking periodontal disease with functional impairments in geriatric patients.

## Conclusion

This study suggests a correlation between geriatric assessments, including the Tinetti Test, handgrip strength, and the MMSE, and the periodontal condition as described by clinical parameters such as CAL, API, and BOP. Among these, the Tinetti Test appears to be the most effective predictor of periodontal health. The findings highlight the need for targeted preventive strategies and equitable, barrier-free access to dental care for hospitalised patients, addressing the periodontal health needs of this vulnerable patient group.

## Supplementary information

Below is the link to the supplementary information.

Supplementary Material 1 (DOCX 64.3 KB)

## Data Availability

The datasets used and/or analyzed during the current study available from the corresponding author on reasonable request.
