# Supplementary material for: Association between periodontal and geriatric health: a cross-sectional study
Source: Clin Oral Investig. 2026 May 30;30(6):263. doi: 10.1007/s00784-026-06938-0 (PMC13222293; doi:10.1007/s00784-026-06938-0)
Supplement: Supplementary file 1 — Supplementary Material 1 (DOCX 64.3 KB) [file 784_2026_6938_MOESM1_ESM.docx]

**Supplementary**

**Table S1: Characteristics of the study population, oral aspects**

| **Variables** | | **n (%)** |
| --- | --- | --- |
| **Dental prosthesis care** | Own teeth | 22 (43.1) |
|  | Own teeth + denture(s) | 25 (49.0) |
|  | Denture(s) | 4 (7.8) |
| **Cleaning behavior** | Independent | 48 (94.1) |
|  | With help | 3 (5.9) |
| **Frequency** | 0/d | 2 (3.9) |
|  | 1/d | 10 (19.6) |
|  | 2/d | 24 (47.1) |
|  | 3/d | 15 (29.04) |
| **Cleaning utensils** | Manual toothbrush | 28 (54.9) |
|  | Manual + Interdental care | 4 (7.8) |
|  | Electric toothbrush | 11 (21.6) |
|  | Electric + Interdental care | 8 (15.7) |
| **Ability to chew** | Yes | 44 (86.3) |
|  | No | 7 (13.7) |
| **Xerostomia** | Yes | 36 (70.6) |
|  | No | 15 (29.4) |
| **Pain** | Yes | 1 (2.0) |
|  | No | 50 (98.0) |

**Table S2.1: Association of CAL and sociodemographic data**

| **Correlated variable** | | **CAL (mean ± SD)** | **Comparison** | **Regression coefficient** | **Std-Error** | **p-value** |
| --- | --- | --- | --- | --- | --- | --- |
| **Sex** | Male | 3.74±1.43 | - | - | - | - |
|  | Female | 3.77±1.48 | Female vs. male | -0.028 | 0.415 | 0.945 |
| **Marital status** | Married | 3.91±1.73 | Married vs. single | -0.284 | 0.642 | 0.658 |
|  | Single | 4.19±1.76 | - | - | - | - |
|  | Widowed | 3.52±1.53 | Widowed vs. single | -0.677 | 0.655 | 0.301 |
| **Living environment** | Alone | 3.92±1.59 | - | - | - | - |
|  | Alone with help | 3.52±1.19 | Alone with help vs. alone | -0.402 | 0.383 | 0.293 |
| **Care level** | 0 | 3.87±1.56 | - | - | - | - |
|  | 1 | 3.44±1.69 | 1 vs. 0 | -0.435 | 0.739 | 0.556 |
|  | 2 | 3.85±1.53 | 2 vs. 0 | -0.022 | 0.546 | 0.968 |
|  | 3 | 3.55±1.0 | 3 vs. 0 | -0.323 | 0.444 | 0.467 |
|  | ≥ 4 | - | ≥ 4 vs. 0 | -0.653 | 0.295 | 0.027** |
| **BMI** | Underweight | 4.36±3.0 | - | - | - | - |
|  | Normal weight | 3.5±3.0 | Normal weight vs. underweight | -0.861 | 1.443 | 0.551 |
|  | Overweight | 4.0±1.46 | Overweight vs. underweight | -0.349 | 1.45 | 0.81 |
|  | Obesity | 3.37±1.41 | Obesity vs. underweight | -0.985 | 1.513 | 0.551 |
| **Diet** | Soft food | 1n | - | - | - | - |
|  | Mixed food | 4.93±2.46 | Mixed food vs No food | 1.705 | 0.918 | 0.063 |
|  | Wholefood | 3.62±1.23 | Wholefood vs No food | 0.396 | 0.183 | 0.031* |

**Table S2.2: Association of CAL and oral health**

| Correlated variable | | CAL (Mean ± SD) | Comparison | Regression coefficient | Std-Error | p-value |
| --- | --- | --- | --- | --- | --- | --- |
| Dysphagia index | No Dysphagia | 3.74±1.33 | - | - | - | - |
|  | Dysphagia | 4.09±3.24 | Dysphagia vs no Dysphagia | -0.351 | 1.544 | 0.82 |
| Dependency | Independent | 3.60±1.21 | - | - | - | - |
|  | Dependent | 6.30±2-67 | Dependent vs Independent | 2.700 | 1.272 | 0.34 |
| Brushing frequency | 0/d | 7.85±0.22 | - | - | - | - |
|  | 1/d | 3.69±1.2 | 1/d vs 0/d | -4.153 | 0.376 | <0.001*** |
|  | 2/d | 3.5±1.18 | 2/d vs 0/d | -4.350 | 0.26 | <0.001*** |
|  | 3/d | 3.69±1.32 | 3/d vs 0/d | -4.153 | 0.376 | <0.001*** |
| Cleaning utensils | Manual toothbrush | 3.99±1.61 | - | - | - | - |
|  | Manual toothbrush + Interdental care | 4.23±1.66 | Manual toothbrush + Interdental care vs Manual toothbrush | 0.243 | 0.78 | 0.755 |
|  | Electric toothbrush | 3.45±0.96 | Electric toothbrush vs Manual toothbrush | -0.537 | 0.409 | 0.189 |
|  | Electric toothbrush + Interdental care | 3.16±1.19 | Electric toothbrush + Interdental care vs Manual toothbrush | -0.828 | 0.497 | 0.96 |
| Xerostomia | Yes | 3.52±1.43 | - | - | - | - |
|  | No | 4.35±1.35 | No vs Yes | 0.833 | 0.411 | 0.43 |
| Acceptable chewing capacity | Yes | 3.65±1.39 | - | - | - | - |
|  | No | 4.45±1.73 | No vs Yes | 0.802 | 0.64 | 0.211 |
| Pain | Yes | - | - | - | - | - |
|  | No | 3.8±1.44 | No vs Yes | 1.91 | 0.201 | <0.001*** |
| Staging | Stage I | - | - | - | - | - |
|  | Stage II | - | - | - | - | - |
|  | Stage III | - | Stage III vs Stage II | 1.626 | 0.281 | <0.001*** |
|  | Stage IV | - | Stage IV vs Stage II | 2.262 | 0.315 | <0.001*** |

**Table S3.1: Association of PPD and sociodemographic data**

| **Correlated variable** | | **PPD (Mean ± SD)** | **Comparison** | **Regression coefficient** | **Std.-Error** | **p-value** |
| --- | --- | --- | --- | --- | --- | --- |
| **Sex** | Male | 2.55±0.68 | - | - | - | - |
|  | Female | 2.61±0.98 | Female vs. male | 0.057 | 0.228 | 0.803 |
| **Marital status** | Married | 2.59±0.57 | Married vs. single | -0.448 | 0.471 | 0.342 |
|  | Single | 3.04±1.37 | - | - | - | - |
|  | Widowed | 2.44±0.85 | Widowed vs. single | -0.6 | 0.483 | 0.214 |
| **Living environment** | Alone | 2.72±0.94 | - | - | - | - |
|  | Alone with help | 2.37±0.72 | Alone with help vs. alone | -0.349 | 0.2289 | 0.127 |
| **Care level** | 0 | 2.72±1.02 | - | - | - | - |
|  | 1 | 2.46±0.79 | 1 vs. 0 | -0.256 | 0.368 | 0.486 |
|  | 2 | 2.29±0.63 | 2 vs. 0 | -0.426 | 0.27 | 0.113 |
|  | 3 | 2.64±0.7 | 3 vs. 0 | -0.076 | 0.3001 | 0.799 |
|  | ≥ 4 | 1n | ≥ 4 vs. 0 | -0.586 | 0.192 | 0.002** |
| **BMI** | Underweight | 2.48±1.45 | - | - | - | - |
|  | Normal weight | 2.43±0.70 | Normal weight vs. underweight | -0.05 | 0.703 | 0.794 |
|  | Overweight | 2.8±0.99 | Overweight vs. underweight | 0.313 | 0.715 | 0.662 |
|  | Obesity | 2.3±0.43 | Obesity vs. underweight | -0.183 | 0.704 | 0.794 |
| **Diet** | Soft food | 1n | - | - | - | - |
|  | Mixed food | 1.69±1.65 | Mixed food vs No food | 1.295 | 0.615 | 0.035* |
|  | Wholefood | 2.48±0.67 | Wholefood vs No food | 0.350 | 0.1 | <0.001*** |

**Table S3.2: Association of PPD and oral health**

| Correlated variable | | PPD (Mean ± SD) | Comparison | Regression coefficient | Std-Error | p-value |
| --- | --- | --- | --- | --- | --- | --- |
| Dysphagia index | No Dysphagia | 2.6±0.82 | - | - | - | - |
|  | Dysphagia | 2.31±1.73 | Dysphagia vs no Dysphagia | 0.295 | 0.825 | 0.721 |
| Dependency | Independent | 2.48±0.67 | - | - | - | - |
|  | Dependent | 4.19±2.1 | Dependent vs Independent | 1.71 | 0.454 | <0.001*** |
| Brushing frequency | 0/d | 5.23±1.56 | - | - | - | - |
|  | 1/d | 2.64±0.63 | 1/d vs 0/d | -2.587 | 0.520 | <0.001*** |
|  | 2/d | 2.39±0.62 | 2/d vs 0/d | -2.836 | 0.494 | <0.001*** |
|  | 3/d | 2.5 | 3/d vs 0/d | -2.587 | 0.505 | <0.001*** |
| Cleaning utensils | Manual toothbrush | 2.82±0.98 | - | - | - | - |
|  | Manual toothbrush + Interdental care | 2.49±0.56 | Manual toothbrush + Interdental care vs Manual toothbrush | -0.33 | 0.304 | 0.278 |
|  | Electric toothbrush | 2.3±0.46 | Electric toothbrush vs Manual toothbrush | -0.515 | 0.225 | 0.022* |
|  | Electric toothbrush + Interdental care | 2.21±0.87 | Electric toothbrush + Interdental care vs Manual toothbrush | -0.612 | 0.340 | 0.072 |
| Xerostomia | Yes | 2.49±0.93 | - | - | - | - |
|  | No | 2.80±0.69 | No vs Yes | 0.312 | 0.23 | 0.174 |
| Acceptable chewing capacity | Yes | 2.48±0.73 | - | - | - | - |
|  | No | 3.21±1.42 | No vs Yes | 0.724 | 0.336 | 0.031* |
| Pain | Yes | 1n | - | - | - | - |
|  | No | 2.60±0.87 | No vs Yes | 1.014 | 0.122 | <0.001*** |
| Staging | Stage I | - | - | - | - | - |
|  | Stage II | 1.54±0.42 | - | - | - | - |
|  | Stage III | 2.51±0.54 | Stage III vs Stage II | 0.977 | 0.191 | <0.001*** |
|  | Stage IV | 2.94±0.88 | Stage IV vs Stage II | 1.403 | 0.139 | <0.001*** |

**Table S4.1: Association of BOP and sociodemographic data**

| **Correlated variable** | | **BOP (Mean ± SD)** | **Comparison** | **Regression coefficient** | **Std.-Error** | **p-value** |
| --- | --- | --- | --- | --- | --- | --- |
| **Sex** | Male | 82.84±21.54 | - | - | - | - |
|  | Female | 73.53±28.63 | Female vs. male | -9.311 | 6.926 | 0.179 |
| **Marital status** | Married | 82.11±22.04 | Married vs. single | -6.139 | 8.203 | 0.454 |
|  | Single | 88.25±19.55 | - | - | - | - |
|  | Widowed | 69.72±29.62 | Widowed vs. single | -18.53 | 8.689 | 0.033* |
| **Living environment** | Alone | 78.10±26.59 | - | - | - | - |
|  | Alone with help | 75.30±26.65 | Alone with help vs. alone | -2.79 | 7.470 | 0.708 |
| **Care level** | 0 | 79.44±26.91 | - | - | - | - |
|  | 1 | 67.60±31.41 | 1 vs. 0 | -11.844 | 13.554 | 0.382 |
|  | 2 | 69.90±30.36 | 2 vs. 0 | -9.544 | 10.431 | 0.360 |
|  | 3 | 83.38±18.11 | 3 vs. 0 | 3.931 | 7.855 | 0.617 |
|  | ≥ 4 | 1n | ≥ 4 vs. 0 | - | - | - |
| **BMI** | Underweight | 69.00±42.93 | - | - | - | - |
|  | Normal weight | 75.63±26.39 | Normal weight vs. underweight | 6.632 | 21.078 | 0.753 |
|  | Overweight | 79.35±26.55 | Overweight vs. underweight | 10.348 | 20.949 | 0.621 |
|  | Obesity | 76.33±23.33 | Obesity vs. underweight | 7.333 | 22.026 | 0.739 |
| **Diet** | Soft food | 1n | - | - | - | - |
|  | Mixed food | 79.±31.52 | Mixed food vs No food | 1.0 | 11.747 | 0.741 |
|  | Wholefood | 76.7±26.33 | Wholefood vs No food | -1.295 | 3.924 | 0.932 |

**Table S4.2: Association of BOP and oral health**

| Correlated variable | | BOP (Mean ± SD) | Comparison | Regression coefficient | Std-Error | p-value |
| --- | --- | --- | --- | --- | --- | --- |
| Dysphagia index | No Dysphagia |  | - | - | - | - |
|  | Dysphagia |  | Dysphagia vs no Dysphagia | 15.229 | 22.408 | 0.497 |
| Dependency | Independent | 76.02±26.78 | - | - | - | - |
|  | Dependent | 92.67±12.70 | Dependent vs Independent | 16.646 | 7.105 | 0.019* |
| Brushing frequency | 0/d | 100±0 | - | - | - | - |
|  | 1/d | 81.60 ± 27.59 | 1/d vs 0/d | -18.4 | 8.276 | 0.026 |
|  | 2/d | 74.38 ± 26.32 | 2/d vs 0/d | -25.625 | 5.26 | <0.001*** |
|  | 3/d | 75.07 ± 27.43 | 3/d vs 0/d | -24.933 | 6.842 | <0.001*** |
| Cleaning utensils | Manual toothbrush | 82.14±23.86 | - | - | - | - |
|  | Manual toothbrush + Interdental care | 65.25±34.36 | Manual toothbrush + Interdental care vs Manual toothbrush | -16.893 | 15.521 | 0.276 |
|  | Electric toothbrush | 75.91±20.45 | Electric toothbrush vs Manual toothbrush | -6.234 | 7.361 | 0.397 |
|  | Electric toothbrush + Interdental care | 66.38±36.84 | Electric toothbrush + Interdental care vs Manual toothbrush | -15.768 | 12.962 | 0.224 |
| Xerostomia | Yes | 72.19±27.01 | - | - | - | - |
|  | No | 88.53±21.47 | No vs Yes | 16.339 | 6.955 | 0.019* |
| Acceptable chewing capacity | Yes | 75.14±27.47 | - | - | - | - |
|  | No | 88.71±14.31 | No vs Yes | 13.578 | 6.47 | 0.036* |
| Pain | Yes | 1n | - | - | - | - |
|  | No | 78.20±25.71 | No vs Yes | 61.2 | 3.529 | <0.001*** |
| Staging | Stage I | 0n | - | - | - | - |
|  | Stage II | 38.13±22.36 | - | - | - | - |
|  | Stage III | 80.75±19.42 | Stage III vs Stage II | 42.625 | 8.762 | <0.001*** |
|  | Stage IV | 86.30±20.57 | Stage IV vs Stage II | 48.171 | 8.352 | <0.001*** |

**Table S5.1: Association of API and sociodemographic data**

| **Correlated variable** | | **API (mean ± SD)** | **Comparison** | **Regression coefficient** | **Std-Error** | **p-value** |
| --- | --- | --- | --- | --- | --- | --- |
| **Sex** | Male | 85.74±28.14 | - | - | - | - |
|  | Female | 79.66±27.26 | Female vs. male | -6.081 | 7.87 | 0.44 |
| **Marital status** | Married | 89.94±18.66 | Married vs. single | 13.069 | 12.438 | 0.293 |
|  | Single | 76.88±35.32 | - | - | - | - |
|  | Widowed | 77.76±29.79 | Widowed vs. single | 0.885 | 13.059 | 0.946 |
| **Living environment** | Alone | 78.45±20.40 | - | - | - | - |
|  | Alone with help | 87.3±23.91 | Alone with help vs. alone | 8.848 | 7.358 | 0.229 |
| **Care level** | 0 | 80.59±26.47 | - | - | - | - |
|  | 1 | 84.60±34.44 | 1 vs. 0 | 4.007 | 14.653 | 0.784 |
|  | 2 | 80.0±35.18 | 2 vs. 0 | -0.593 | 11.678 | 0.960 |
|  | 3 | 84.88±21.15 | 3 vs. 0 | 4.282 | 8.597 | 0.618 |
|  | ≥ 4 | 1n | ≥ 4 vs. 0 | 19.407 | 4.999 | <0.001*** |
| **BMI** | Underweight | 73.67±45.61 | - | - | - | - |
|  | Normal weight | 79.21±29.76 | Normal weight vs. underweight | 5.544 | 22.505 | 0.805 |
|  | Overweight | 83.39±26.33 | Overweight vs. underweight | 9.725 | 22.162 | 0.661 |
|  | Obesity | 89.0±18.41 | Obesity vs. underweight | 15.333 | 22.569 | 0.497 |
| **Diet** | Soft food | 1n | - | - | - | - |
|  | Mixed food | 77.5±26.77 | Mixed food vs No food | -22.5 | 9.977 | 0.024* |
|  | Wholefood | 82.11±28.0 | Wholefood vs No food | -17.886 | 4.173 | <0.001*** |

**Table S5.2: Association of API with oral health**

| Correlated variable | | API (Mean ± SD) | Comparison | Regression coefficient | Std-Error | p-value |
| --- | --- | --- | --- | --- | --- | --- |
| Dysphagia index | No Dysphagia | 82.52±26.44 | - | - | - | - |
|  | Dysphagia | 72.33±47.92 | Dysphagia vs no Dysphagia | 10.188 | 22.903 | 0.656 |
| Dependency | Independent | 80.79±27.94 | - | - | - | - |
|  | Dependent | 100±0 | Dependent vs Independent | 19.208 | 3.992 | <0.001*** |
| Brushing frequency | 0/d | 100±0 | - | - | - | - |
|  | 1/d | 82.3±30.71 | 1/d vs 0/d | -17.7 | 9.213 | 0.055 |
|  | 2/d | 81.54±27.89 | 2/d vs 0/d | -18.458 | 5.573 | <0.001*** |
|  | 3/d | 79.87±27.58 | 3/d vs 0/d | -20.133 | 6.879 | 0.003** |
| Cleaning utensils | Manual toothbrush | 89.75±22.51 | - | - | - | - |
|  | Manual toothbrush + Interdental care | 76.5±33.96 | Manual toothbrush + Interdental care vs Manual toothbrush | -13.25 | 15.285 | 0.386 |
|  | Electric toothbrush | 73.09±25.26 | Electric toothbrush vs Manual toothbrush | -16.659 | 8.379 | 0.047* |
|  | Electric toothbrush + Interdental care | 69.38±38.31 | Electric toothbrush + Interdental care vs Manual toothbrush | -20.375 | 13.34 | 0.127 |
| Xerostomia | Yes | 81.19±27.75 | - | - | - | - |
|  | No | 83.67±27.69 | No vs Yes | 2.472 | 8.278 | 0.765 |
| Acceptable chewing capacity | Yes | 80.43±28.82 | - | - | - | - |
|  | No | 91.29±14.91 | No vs Yes | 10.854 | 6.757 | 0.108 |
| Pain | Yes | 1n | - | - | - | - |
|  | No | 82.88±26.88 | No vs Yes | 48.88 | 3.763 | <0.001*** |
| Staging | Stage I | 0n | - | - | - | - |
|  | Stage II | 64.63±29.9 | - | - | - | - |
|  | Stage III | 84.88±28.25 | Stage III vs Stage II | 20.25 | 12.021 | 0.092 |
|  | Stage IV | 85.3±25.33 | Stage IV vs Stage II | 20.671 | 10.984 | 0.060 |
